# Gonorrhœa with Shot-Gun Treatment

**Published:** 1891-10

**Authors:** C. M. Boger

**Affiliations:** Parkersburg, W. Va.


### GONORRHŒA WITH SHOT-GUN TREATMENT.

PARKERSBURG, W. VA., July 13th, 1891.

EDITOR OF THE HOMŒOPATHIC PHYSICIAN:

I like your journal very well, but it must be confessed that in looking over the last six numbers I find some things that astonish me not a little, for instance, Dr. Allen's treatment of gonorrhœa. Not long since a brakeman working in the railroad yards here was caught between the bumpers of two cars and slightly injured internally, the principal symptoms referring to the spleen. He recovered rapidly, although he remained extremely weak for some little time. One day he sent for me in haste, and I found a new trouble—the prostate gland had swelled to about the size of a walnut and was very painful. He confessed to having had gonorrhœa, and using what he called the "shot gun" on it. There was still some discharge from the meatus; the gland continued to swell. The next day the inflammation extended into the scrotum, and on the third day the scrotum would have filled any ordinary man's hat; it was tense and of a deep red hue. His sufferings were intense and he insisted on immediate operative procedure. This I declined to do, so he called in an allopath in consultation, who also declined to operate, and he was allowed to go until the next morning, when we again saw him together. Fluctuation being now distinct, but deep in the perinæum, we decided to make an incision, which I accordingly did on the left of the median line. At once there followed about a quart of the vilest-smelling, broken-down, and necrotic tissue I ever saw. It was of a dark chocolate color and had the odor of a bushel of rotten onions. The stench was almost unbearable, sickening both myself and colleague. We elevated the scrotum, and for three days this discharge continued with but little interruption, not so copiously as at first, of course. In those few days our patient lost thirty-five pounds. After the incision he received Crotal-horrid. Nevertheless he did recover, although the allopath said his chances for death were *very bright*. He was at the end of all this a mere skeleton of his former self; the entire contents of the scrotum had softened and pulpified with the exception of a mere caricature of the former testicles, and now he is practically a eunuch. It is not probable that this kind of a case occurs very frequently, but it is a most instructive one to all homœopathicians, and were it possible, I would like to have an immense clinic with all homœopathic "shot-gun" prescribers there, show them this patient, and ask them what they think of their treatment for gonorrhœa. Not every prescriber, I take it, receives such a lesson as this, but allow me to state that my patients have received their last injection for gonorrhœa. I never looked upon the procedure with much favor, but did occasionally allow it when the patient seemed desirous of that treatment, and had frequently seen orchitis result.

My note-book contains several records of cures that I am proud of, but as yet some of them lack the seasoning of time, which should never be overlooked, and the hasty reporting of cures is, I am satisfied, to be carefully avoided. Here is one that is fully matured, and I send it not so much for its striking features as from the fact that it is a representative of a large class of patients who come to our offices with illy-defined and obscure symptoms, but who nevertheless must have relief and are often exceedingly difficult to prescribe for.

D. O. came to my office February 2d, states his occupation as that of contractor, and sometimes does very heavy lifting. Age about forty; wants relief for the following symptoms:

"Dull pain below right nipple, going into right abdomen; worse by motion. Chronic nasal catarrh, nostrils open alternately. Constant ringing in left ear; worse on going to bed at night; better in morning; first caused by diving, which he followed as an occupation years ago. Constipation; stool small, unsatisfactory; after stool, burning in anus. After rising in morning, dull headache in forehead; better from exercise. Heart misses a beat now and then, about tenth or twelfth. After coition urine smells strong, like horse's urine. Water-brash after eating; better from coffee; worse from salt meat or beef. Sensation of a lump in stomach after meals. Chews tobacco excessively. Stopped the tobacco and gave him Sulph.$^{200}$, four powders, followed by Sac-lac. February 11th, all symptoms better, except No. 1. ℞ Sac-lac. March 5th, still improving, ℞ Sac-lac. July 10th, remains well to date."

Fraternally yours,

C. M. Boger.

